# Combination of transcatheter arterial chemoembolization and portal vein embolization for patients with hepatocellular carcinoma: a review

**DOI:** 10.1186/s12957-021-02401-4

**Published:** 2021-10-01

**Authors:** Zhiying Shao, Xin Liu, Chanjuan Peng, Liping Wang, Dong Xu

**Affiliations:** Department of Interventional Ultrasound, The Cancer Hospital of the University of Chinese Academy of Sciences (Zhejiang Cancer Hospital), Institute of Basic Medicine and Cancer (IBMC), Chinese Academy of Sciences , No. 1 East Banshan Road, Gongshu District, Hangzhou, 310022 People’s Republic of China

**Keywords:** Hepatocellular carcinoma, Portal vein chemoembolization, Portal vein embolization, Transcatheter arterial chemoembolization

## Abstract

**Background:**

Transcatheter arterial chemoembolization has been widely used in patients with hepatocellular carcinoma. However, double blood supply and the existence of portal vein tumor thrombus influence the efficacy of transcatheter arterial chemoembolization.

**Main body:**

Theoretically, portal vein embolization combined with transcatheter arterial chemoembolization may bring a breakthrough in the therapeutic effect of hepatocellular carcinoma. The feasibility, efficacy, long-term survival benefits, and side effects of the combined treatment have been explored in previous studies. Chemotherapeutic agents may also be added in the portal vein embolization procedure to further improve the treatment response.

**Conclusion:**

In this study, we review the existing data and studies on the combined treatment in patients with hepatocellular carcinoma and provide an overall view of the strategy.

## Background

Hepatocellular carcinoma (HCC) is the sixth most common cancer with rising cancer-related mortality worldwide [1, 2]. At present, the main means of radical treatment include surgical resection, liver transplantation, and thermal ablation. However, due to advanced stage at diagnosis, comorbidity, poor hepatic reserve function, and/or lack of suitable donor livers, most of the patients with HCC are not candidates for radical therapy [3, 4]. Even after curative resection, the prognosis for these patients is still poor due to the high incidence of liver failure, the existence of portal vein thrombosis, or microscopic tumor thrombosis [5–7].

Given the limitations of locoregional treatment modalities, transcatheter arterial chemoembolization (TACE) is widely used as a postoperative procedure to improve treatment response [3, 8]. TACE is recommended in the current treatment guidelines for Barcelona clinic liver cancer (BCLC) stage B HCC patients [9, 10]. In real-life management, TACE is actually extensively applied in clinical practice. The global HCC BRIDGE study retrospectively collected 18,031 cases from 14 countries, and its results indicated that TACE was wildly applied as the primary treatment for HCC across all stages in North America, Europe, China, and South Korea [11]. However, long-term clinical experience has shown that due to the rich blood supply of HCC, the production of new blood vessels after TACE, and the establishment of collateral circulation, TACE is insufficient to achieve the desired therapeutic effect, and its long-term benefit of reducing tumor recurrence has been questioned in some studies [12–15].

With a variety of perfusion techniques, some scholars have found that 57.7% of liver cancer nodules are nourished by both hepatic artery and portal vein, whereas 24.4% only have hepatic artery blood supply and 17.8% only have portal vein blood supply [16, 17]. Meanwhile, the central part of HCC is mainly supplied by the hepatic artery, but the marginal area where the tumor is actively growing and infiltrating is mainly supported by the portal vein. Moreover, for non-capsule HCC, the proportion of the portal vein blood supply exceeds that of the hepatic artery [18]. Given the facts above, the efficacy of TACE may be unsatisfactory for HCC nodules mainly or only nourished by the portal vein [19]. Portal vein tumor thrombus (PVTT) is another important consideration that may influence the efficacy of TACE. It occurs in about 10% to over 60% of HCC cases [20]. The existence of PVTT may cause portal hypertension and metastasis, leading to liver failure and poor prognosis [21, 22].

The safety and efficacy of portal vein embolization (PVE) for reliably producing significant hypertrophy in the future remnant liver (FRL) prior to the planned resection have been well-established [23]. For patients with unresectable HCC, sequential TACE + PVE may increase FRL hypertrophy and tumor necrosis rate, leading to prolonged survival time in HCC patients [24–26]. From a pathophysiological perspective, chemotherapeutic drugs and lipiodol in the hepatic artery can return to the portal vein after the TACE procedure and then act on the PVTT. PVE can embolize the blood supply of the primary tumor in the portal vein and maintain a high local concentration of the chemotherapeutic drugs [18]. In the previous studies, the incidence rate of TACE + PVE–induced complete tumor necrosis was 80%, which was significantly higher compared with that in the treatment of TACE alone (50%) and PVE alone (5%) [27, 28]. Animal model experiments also confirmed the distinguished efficacy [29, 30].

**Fig. 1:**
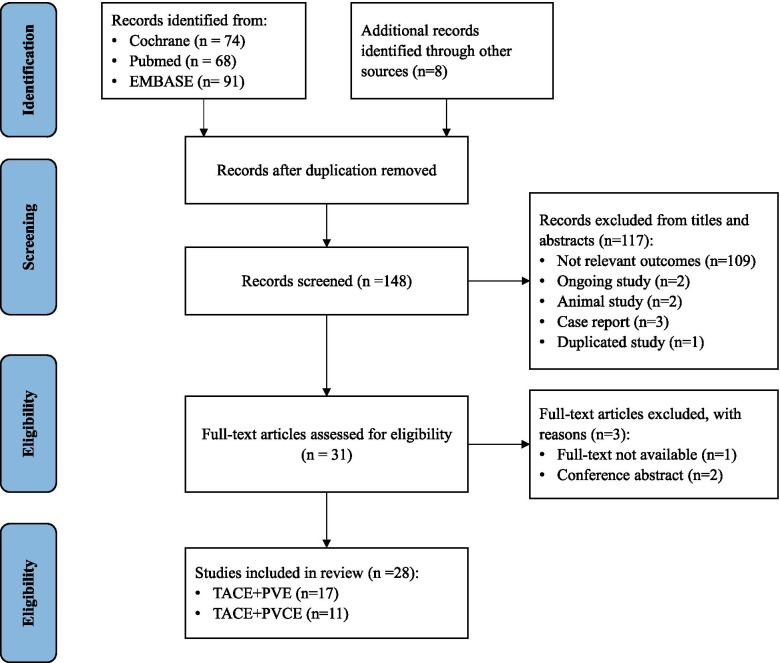
The flow chart represents the screening process

As of August 11, 2021, studies were identified through a search of PubMed, Embase, and Cochrane Library. The retrieval strategy included title, abstract, and keywords. We combined the terms such as “transcatheter arterial chemoembolization” or “TACE” with “portal vein embolization” or “PVE”; “portal vein chemoembolization” or “PVCE”. The included studies only aimed at HCC. No language limitations were imposed in our search. Figure 1 shows the detailed screening process, and Table 1 listed the basic characteristics of all the articles eventually identified in this review. This study aimed to qualitatively evaluate the feasibility and efficacy of dual-embolization procedure, report recent advances of the combined treatment, adverse effects that may occur, as well as other potential improvements in the future.

**Table 1:** The basic characteristics of identified studies in this review

Study	Country	Year	Type	Disease	Study arms	NP	Treatment phase
Taku et al. [25]	Japan	2004	Retrospective	HCC with chronic liver disease	TACE + PVE	17	Preoperative treatment
Ogata et al. [28]	France	2006	Retrospective	HCC with chronic liver disease	TACE + PVE vs. PVE	36	Preoperative treatment
Imamura et al. [31]	Japan	2008	Prospective	HCC	TACE + PVE	45	Preoperative treatment
Yoo et al. [32]	Korea	2011	Retrospective	HCC	TACE + PVE vs. PVE	135	Preoperative treatment
Xu et al. [33]	China	2014	Retrospective	HCC	TACE + PVE	37	Preoperative treatment
Choi et al. [34]	Korea	2015	Retrospective	HCC	TACE + PVE	113	Preoperative treatment
Ronot et al. [35]	France	2016	Retrospective	HCC	TACE + PVE	54	Preoperative treatment
Peng et al. [36]	China	2017	Prospective	Large HCC	TACE + PVE vs. PVE	13	Preoperative treatment
Terasawa et al. [37]	France	2020	Prospective	Large HCC	Simultaneous TACE + PVE vs. sequential TACE + PVE vs. PVE	55	Preoperative treatment
Zhang et al. [38]	China	2020	Retrospective	Large HCC	TACE + PVE vs. TACE	51	Preoperative treatment
Park et al. [39]	Korea	2020	Retrospective	HCC	TACE + PVE vs. PVE vs. TACE vs. naïve control	205	Preoperative treatment
Peng et al. [40]	America	2012	Retrospective	HCC with cirrhosis	TACE + PVE vs. PVE vs. TACE	56	Preoperative treatment
Sommacale et al. [41]	France	2014	Retrospective	HCC	TACE + PVE vs. PVE	24	Preoperative treatment
Mao et al. [42]	China	2002	perspective	HCC	TACE + PVE	209	Palliative treatment
Kang et al. [43]	Korea	2009	Retrospective	HCC	TACE + PVE	25	Salvage treatment
Tan et al. [18]	China	2014	Retrospective	HCC with PVTT	TACE + PVE vs. TACE	116	Palliative treatment
Okabe et al. [44]	Japan	2012	Retrospective	HCC	TACE + PVE vs. TACE	39	Palliative treatment
Wu et al. [45]	China	2003	Retrospective	HCC	TACE + PVCE vs. PVCE	176	Palliative treatment
Liang et al. [46]	China	2009	Prospective	HCC	TACE + PVCE vs. TACE	32	Palliative treatment
He et al. [47]	China	2010	Prospective	PLC	TACE + PVCE vs. TACE	48	Palliative treatment
Zhang et al.[48]	China	2012	Prospective	HCC with PVTT	TACE vs. PVE vs. TACE + PVCE	87	Postoperative treatment
Guo et al. [49]	China	2011	Prospective	HCC	TACE + PVCE vs. TACE	36	Palliative treatment
Jia et al. [50]	China	2017	Prospective	HCC	TACE + PVCE vs. TACE	48	Palliative treatment
Yin et al. [51]	China	2007	Prospective	HCC	TACE + PVCE	54	Palliative treatment
Li et al. [52]	China	2006	Prospective	HCC	TACE + PVCE vs. TACE vs. naïve control	131	Postoperative treatment
Dai et al. [7]	China	2019	Retrospective	HCC with PVTT	TACE + PVCE vs. TACE	119	Postoperative treatment
He et al. [53]	China	2018	Prospective	HCC	TACE + PVCE vs. TACE	133	Palliative treatment
Zhao et al. [54]	China	2009	Retrospective	HCC with PVTT	TACE + PVCE vs. TACE	48	Palliative treatment

*HCC* Hepatocellular carcinoma, *PVTT* Portal vein tumor thrombus, *TACE* Transcatheter arterial chemoembolization, *PVE* Transcatheter arterial chemoembolization, *PVCE *Portal vein chemoembolization, *NP* Number of patients

### Treatment procedure

A conventional approach (cTACE) was used in all studies identified. cTACE included an assessment by conventional mesenteric arteriography, an intra-arterial injection of a mixture of chemotherapy and emulsified iodized oil and a consolidated embolization achieved by injection of gelatin sponge or polyvinyl alcohol particles. Drug-eluting bead TACE (DEB-TACE) was also applied in few recent studies [35, 40].

The traditional PVE approach was selectively catheterizing a portal branch contralateral to the tumor under ultrasonographic guidance. Then, a portography was performed and a guidewire was placed ipsilateral to the tumor. Embolic material varies depending on operator preference. Later studies proposed that the percutaneous puncture route could be either ipsilateral or contralateral according to tumor location. Compared to the traditional contralateral approach, ipsilateral one is more popular now due to the lower risk of damage to the FRL. However, for patients with a large tumor located in the puncture route, the risk of tumor implantation may be higher [55, 56]. Recently, novel strategies such as trans-splenic and trans-jugular approaches are under investigated [57, 58]. The safety has been confirmed in a few studies and may be the alternative routes for patients with a challenging trans-hepatic route [59–61].

PVCE was performed using the same technique as PVE. In general, the chemotherapy regimen was the same as TACE, but with reduced dosage in some studies [42, 51].

### TACE combined with PVE in patients with HCC

The application of PVE was documented in as early as the 1980s [62]. It was initially performed for patients with hilar bile duct carcinoma. Gradually, given the simplicity of technology, its indications have been expanded to HCC and become a standard preoperative intervention for patients with HCC globally [63, 64]. Nevertheless, the compensatory increase of the hepatic arterial flow resulting from PVE may lead to rapid ipsilateral tumor growth as well as insufficient FRL hypertrophy [38]. The association live partition and portal vein ligation for staged hepatectomy is an innovative procedure to rapidly increase FRL volume, but at the expense of large injury and high incidence of postoperative complications.[65]. For patients with HCC planning to receive liver resection, TACE followed by PVE is applied to increase tumor necrosis, boost FRL hypertrophy, and prevent tumor progression during the gap between PVE and planned surgery [31, 66, 67].

Most of the identified studies were in small size. However, all of them affirmed the safety and efficacy of such double occlusion before planned resection, even in chronic liver disease cohort [25, 28] and large tumor (≥ 50 mm) cohort [34, 36–38]. Yoo et al. retrospectively compared the clinical outcome of the TACE-PVE procedure (*n* = 71) with PVE alone (*n* = 64) prior to right hepatectomy in patients with HCC. FRL volume was significantly increased in the combined group (7.3% vs. 5.8%, *P* = 0.035). The cumulative recurrence-free survival rate at 10 years was also notably higher in the combined group (56% vs. 24%, *P* = 0.001), leading to a longer overall survival (OS) as well (*P* = 0.028). Moreover, the TACE-PVE procedure may increase the resectability, even for patients classified as BCLC stage B and were not qualified for surgery theoretically. Even when patients are deemed unsuitable for scheduled surgery with various reasons, alternative therapy including repeat TACE is still tolerable [32]. Recently, a similar retrospective study confirmed the efficacy of sequential TACE-PVE. It is the largest published study till now which analyzed the long-term outcome of sequential TACE-PVE in HCC patients who planned to receive right hemihepatectomy. Overall, 109 of 205 patients received TACE-PVE before liver resection, whereas 28 received TACE alone, 38 received PVE alone, and 30 were in the naïve control group. The OS and disease-free survival (DFS) were both significantly higher in the TACE-PVE group (both *P* < 0.001). Notably, although the extra damage of the noncancerous liver parenchyma was minimal in the TACE-PVE group, technical difficulties of surgery were increased requiring close attention to avoid dense inflammatory adhesion and choledochal varices [39]. The only multi-center study from Peng et al. confirmed the feasibility and safety of sequential TACE and PVE in liver malignancies (the majority of patients had HCC). Although a noticeable difference in FRL hypertrophy was not detected in the combined group compared with PVE alone. Greater than 50% tumor necrosis and a longer DFS time were noted among most patients received sequential TACE and PVE treatment [40].

For patients with unresectable HCC, TACE is applied as an effective palliative therapy [68]. However, TACE exhibits great efficacy when performed on the portal embolized areas but not the non-portal embolized ones, which may lead to recurrence especially for large tumors. Sequential TACE + PVE may contribute to complete tumor necrosis and prolonged survival time, even in patients with PVTT [18, 69, 70]. Okabe et al. conducted a small study including 17 patients who received TACE combined with PVE and 22 patients who received TACE only. The long-term survival analysis showed that the intrahepatic recurrence rates in the non-portal-embolized area were much lower in the TACE + PVE group than those in the TACE only group (41.1% vs. 77.3%, 58.8% vs. 81.8%; *P* = 0.027). The 5-year OS was 38.2% in the TACE + PVE group compared with only 8.5% in the TACE only group (*P* = 0.046) [44]. Another small study from Kang et al., instead, failed to show statistically significant survival benefits [43]. Therefore, such single-centered small-scale studies are difficult to provide compelling evidence to support the combined treatment. Mao et al. conducted a randomized-control clinical study to conduct the efficacy and long-term survival rates between TACE only group (*n* = 104) and TACE + PVE group (*n* = 105). Higher complete or partial response rates were detected in patients receiving combined treatment compared with TACE only group (57.2% vs. 37.5%; *P* < 0.01). And the short-term benefit finally transformed into marked prolonged survival time in TACE + PVE group [42]. Still, the study was reported almost two decades ago, the technique and treatment concept were developed up to date, high-quality prospective research is urgently needed to verify the findings.

### TACE combined with PVCE in patients with operative HCC

With mature techniques of PVE, researchers have begun to include the use of chemotherapeutic agents in the PVE procedure, which is called portal vein chemoembolization (PVCE). The efficacy of TACE combined with PVCE in an HCC cohort has been reported in a few scattered small studies [49–51]. Li et al. conducted a relatively high-quality study that enrolled 131 patients with HCC. All the patients were randomly divided into three groups: (1) operation only (group A, *n* = 45), (2) operation plus 3-course TACE (group B, *n* = 39), and (3) operation plus 3-course TACE and 3-course PVCE (group C, *n* = 47). The Kaplan–Meier estimated DFS rates at 1, 3, and 5 years were all significantly higher in group C compared with those in the other groups (*P* < 0.05) [52]. However, such survival benefit was not notable in the subgroup analysis of HCC complicated by PVTT. In this cohort, TACE plus PVCE only benefited patients in the short term (less than 60 months) [3]. The unsatisfactory results may be related to the old operation modalities during the study period (from January 1998 to January 2001). In a recent study, 119 HCC patients with PVTT undergoing surgical treatment from January 2010 to January 2016 were retrospectively analyzed. All the patients underwent placement of an intravenous chemotherapy pump during operation. Among them, 64 patients received postoperative TACE + PVCE, whereas 55 patients only received postoperative TACE. The DFS time of the TACE + PVCE group was twice as much as that of the TACE only group (13.3 months vs. 6.8 months); the OS time was also dramatically prolonged (19.5 months vs. 12.5 months). Therefore, the long-term benefit of TACE + PVCE was reported along with improved operation and postoperative treatment modalities [7].

### TACE combined with PVCE for inoperable HCC

For patients who are not candidates for surgery, retrospective analysis from Wu et al. showed significantly increased response rate in TACE + PVE group but not prolonged survival time [45]. The possible explanation was the study included all the primary liver cancer (PLC) cases not only HCC. Meanwhile, the relatively high dosage of lipiodol in combined group was related to worse liver function injury after treatment which may compromise the long-term survival benefit. He et al. reported a randomized study enrolling 133 patients with advanced HCC. All the patients were not suitable for surgery and were divided into two groups according to treatment: (1) TACE (*n* = 66) and (2) TACE + PVCE (*n* = 67). The overall response rate was significantly higher in the combined therapy group (59% vs. 38%, *P* < 0.01). During the long-term follow-up, the combined therapy group also presented its advantage. The 2-year OS rate was almost twice as much as that of the monotherapy group (60% vs. 37%, *P* < 0.01) [53]. Even if patients failed to get benefit from a single TACE, PVCE can still be performed consequently with other local treatments to improve treatment efficiency, such as radiofrequency ablation. Qian et al. conducted an observational study including 28 cases with HCC who received poor therapeutic efficacy from TACE. Radiofrequency ablation (RFA) followed by immediate PVCE was performed as salvage therapy. No severe complication occurred during or after the treatment, and the thoroughly ablating rate at the end of the 3-month follow-up was 94.4%. The Child–Pugh score of liver function significantly lowered at 3 months after locoregional treatment (*P* < 0.05). This study preliminarily proved the safety and efficacy of such combined treatment. Repeatability is one of the advantages of such local treatment; recurrent or residual disease can still be treated with RFA and PVCE [71]. However, the short-term follow-up period and small sample of the study failed to provide reliable long-term survival data.

### TACE combined with PVE or PVCE for inoperable HCC with PVTT

One of the difficulties of treating inoperable HCC is PVTT. In the past, it was considered that HCC combined with PVTT is an absolute or relative contraindication for TACE. It has been established that PVTT only reduces the blood flow of the portal vein to a certain extent and rarely completely blocks the blood vessels. Furthermore, during the slow process of thrombus formation, the collateral circulation cannot be formed due to the insufficient time [72]. Zhao et al. conducted a small prospective case–control study that enrolled 48 patients with inoperable HCC with PVTT. Overall, 23 of them were treated with TACE combined with PVCE, whereas the remaining patients received TACE alone. The results showed that the rate of the PVTT reduction rate was significantly higher in the TACE + PVCE group and the combined treatment also relieved the gastrointestinal symptoms better (*P* < 0.05). The survival rate also improved during the 1-year follow-up (48% VS. 25%, *P* < 0.05) [54]. Another similar study included 116 HCC patients with PVTT who were treated with TACE alone (*n* = 64) or TACE + PVE (*n* = 52) and followed up for a longer time [18]. The results demonstrated a significant prognostic benefit in the combined therapy group. The subgroup analysis further emphasized the benefits in patients with type-I PVTT in which tumor thrombi were only limited to the partial branches of the portal vein or above it [18, 73].

### Side effects and complications of TACE + PVCE

The chemotherapy agents that are used for TACE and PVCE have rarely changed throughout the last few decades. 5-Fu, mitomycin, Adriamycin, and platinum agents are most commonly used. Only a few early studies focused on the side effects aside from the feasibility and efficacy of TACE combined with PVCE [74, 75]. However, it has been riveting scholars’ attention recently that side effects and complications were more commonly reported in combined group and were relate to postembolization syndrome or chemotherapy. Nausea, vomiting, fever, abdominal discomfort, and liver decompensation were frequently reported [7, 49–53, 76]. Hemorrhage, ascites, and myelosuppression have also been observed in a few studies [7, 52, 76]. Nevertheless, these side effects were transient and mild. The majority of patients recovered within 2 weeks with symptomatic treatment [46, 49, 50]. It is worth noting that PVCE procedure may increase the portal vein pressure and lead to esophageal variceal bleeding. Therefore, patients with severe liver cirrhosis, esophageal varices, portal hypertension, or arteriovenous shunting should be treated with intense caution [47]. Meanwhile, the amount of lipiodol is positively correlated with the degree of liver damage and the efficacy [45, 53]. Although the PVCE procedure is simple and safe, the amount of lipiodol that enters the portal vein is limited, and it is difficult to embolize all the portal vein branches of the tumor but increase liver damage at cross purpose. Further efforts should be made on improve the dual embolization technique for a better balance between efficacy and toxicity.

### Further improvement of chemoembolization

Although TACE has been widely accepted and performed worldwide, conventional TACE (cTACE) procedure has two major defects: (a) local deposition of lipiodol emulsion may fail to achieve satisfactory results, and the cytotoxic effect of chemotherapeutic drugs in tumor tissues decreases with time; (b) the traditional drug carrier is lipiodol, whereas chemotherapeutic drugs are water-soluble, which may cause the rapid release of chemotherapeutic drugs into the bloodstream [77, 78]. Therefore, systemic adverse effects may increase while the local efficacy is reduced. Previous studies have shown that only 20 to 50% of tumor tissues undergo complete necrosis after cTACE treatment (iodine oil + chemotherapy + granule embolization) and the 5-year survival rate is only 9 to 16.2% [68, 79, 80].

A novel type of vascular embolization material, DEB, may solve the problem. The application of the lipiodol-free delivery system can continuously release anti-tumor drugs to increase its local concentration, prolong the action time, reduce peripheral blood concentration, and reduce systemic adverse reactions. In the PRECISION V trial, patients with unresectable HCC were randomized to receive cTACE or DEB-TACE. An equivalent anti-tumor effect was detected in both groups with a significant reduction in serious hepatic toxicity and drug-related side effects in the DEB-TACE group [81, 82]. With super selection, DEB-TACE with a diameter of > 100 μm is superior to cTACE in decreasing the number of procedures, reducing side effects, shortening hospital stay, and improving quality of life, especially for patients with advanced or recurrent disease, Child–Pugh B grade, physical activity level 1, or dual-lobe disease [83, 84].

## Conclusion

In conclusion, TACE combined with PVE or PVCE may be a valuable therapeutic strategy for HCC patients in improving efficacy, preventing recurrence, and prolonging survival time, even in patients. However, hepatic infarction caused by TACE combined with PVE or PVCE may not be tolerated by all patients depending on degree of tumor burden, ability to perform superselective embolization and so forth. Such aggressive treatment is more suitable for patients in relatively good condition. Patients with chronic liver disease or PVTT are not absolutely contraindicated, but intensive pre-treatment evaluation and multidisciplinary discussion should be considered. Large-scale prospective studies are urgently needed to further verify the safety and efficacy of the combined treatment.

## Data Availability

The datasets used and/or analyzed during the current study are available from the corresponding author on reasonable request.

## Abbreviations

HCC: Hepatocellular carcinoma
TACE: Transcatheter arterial chemoembolization
BCLC: Barcelona clinic liver cancer
PVTT: Portal vein tumor thrombus
PVE: Portal vein embolization
FRL: Future remnant liver
DEB: Drug-eluting beads
OS: Overall survival
DFS: Disease-free survival
PVCE: Portal vein chemoembolization
PLC: Primary liver cancer
RFA: Radiofrequency ablation
